# Basic Insights of Lung Ultrasonography in Critical Care Setting

**DOI:** 10.7759/cureus.3702

**Published:** 2018-12-07

**Authors:** Shantanu Singh, Harleen Kaur, Shivank Singh, Imran Khawaja

**Affiliations:** 1 Pulmonary Medicine, Marshall University School of Medicine, Huntington, USA; 2 Neurology, Univeristy of Missouri, Columbia, USA; 3 Internal Medicine, Maoming People's Hospital, Maoming, CHN

**Keywords:** lung ultrasonography, imaging, ultrasound, intensive care unit(icu)

## Abstract

Lung ultrasonography has a tailored diagnostic and therapeutic approach in the critical care setting. Lung ultrasonography in critically ill (LUCI) is a helpful modality for the early detection and assessment of various lung pathologies and guides the management protocol for the same. The aim of this review was to highlight the basics of an ultrasound machine, the fundamentals of a lung ultrasound and the importance of lung artifacts in detecting the anatomy and pathology of the lung disease. In addition, we have also discussed regarding the effective approach to lung ultrasonography through the two protocols: the Bedside Lung Ultrasound in Emergency (BLUE) protocol and the Fluid Administration Limited by Lung Sonography (FALLS) protocol.

## Introduction and background

Point-of-care ultrasonography (POCUS) has gained its utility in critical care medicine during the recent years. Ultrasonography is a rapid, noninvasive, real-time imaging modality that can be used for diagnosis, monitoring the course of the disease and guide the management protocol. Over the past two decades, POCUS is rapidly used to tailor the diagnostic and therapeutic approach in critical illness. The use of an ultrasound machine as a diagnostic tool in medicine was first introduced by a neurologist Karl Dussik in 1942 [1]. The diagnostic application of ultrasound was first described by Frenchman André Dénier [2]. More so, the utility of lung ultrasound in emergency care was introduced by the Fraçois Jardins intensive care team in 1989. Since 1991, POCUS was utilized by intensivists for diagnosis and vascular access in critical care medicine. In recent years, Lung ultrasound in critically ill (LUCI) has gained its popularity in diagnostic and therapeutic approach. The World Interactive Network Focused on Critical Ultrasound (WINFOCUS) has standardized nomenclature, technique and indications to use lung ultrasound in critical care practice in the International Consensus Conference on Lung Ultrasound (ICC-LUS) [3].

The aim of this review was to highlight the fundamentals of lung ultrasound and its utility in critical care medicine for easy evaluation of various lung pathologies.

## Review

Basics of an ultrasound machine

The physics of an ultrasound machine includes the generation of sound waves by piezoelectric crystals in the transducer. These sound waves propagate through various mediums such as tissues, air, blood or bone in the form of mechanical energy. When these sound waves encounter tissues, one or all of the following occur: reflection, scattering, refraction, absorption and attenuation [4].

To understand the concept of reflection, it is important to know about acoustic impedance. The acoustic impedance of a tissue is defined as the product of tissue density and the propagation velocity of the sound waves through that tissue. When the sound waves travel through the interface between two types of tissues, some of these sound waves are reflected back and are known as an echo. The generation of the echo is directly proportional to the acoustic impedance between the two types of tissue. Similarly, the signal brightness is largely dependent on these reflected waves. Hence, a large acoustic impedance difference will result in a bright echogenic signal, and a small acoustic impedance mismatch will result in an echo-poor signal. Most fluids and blood have relatively fewer acoustic impedance and hence produce hypoechoic signals. Air is a poor conductor of sound and hence can result in difficult visualization or artifact. It is impossible to view the structures under the bone, as the bone results in complete reflection of the sound waves. Likewise, the application of coupling gel for imaging purpose is also based on the fact of a large acoustic impedance difference between the air and the skin. Scattering of the sound waves occurs when the size of the tissue is smaller than the encountered ultrasound wavelength. Refraction is the change in the direction of the original sound wave as a result of the propagation of sound waves through various tissues at different velocities. Likewise, the propagation of some of the sound waves through the tissues may result in absorption of this energy. The absorption depends on the frequency of sound waves, the type of tissues and the depth of scanning.

Further, it is important to know that the propagation of ultrasound waves is influenced by the following parameters: frequency, amplitude, period, wavelength and power. Frequency is measured in Hertz (Hz) and is defined as the number of waves passing per second. A period is measured in milliseconds (ms) and is defined as the time taken by one complete wave to pass. Amplitude is defined as the strength of the sound waves and is measured in megapascals (MPa). The power is the total energy of the sound waves and is measured in watts (W) The higher the frequency, the shorter the wavelength and the period of the sound wave. The ultrasound machine works in the range of one to 20 megahertz (MHz). Transducers with a frequency range of 5 to 15 MHz are beneficial to image superficial structures like lung and vascular anatomy, whereas transducers with a frequency of 2 to 5 MHz are used to image deeper tissues like intrabdominal organs.

The common transducers used in ultrasound imaging are linear, phased and curvilinear array type. The linear array probe is used to image the superficial structures and vasculatures. The phased array type is used to image the cardiac domain, and the curvilinear array is used to image the intraabdominal organs [5].

Fundamentals of lung ultrasonography

Lung ultrasonography (LUS) can be a simplified or comprehensive exam and can usually range from 5 to 15 minutes [6]. The LUS is usually preferred in the brightness mode (B-mode) or the motion mode (M-mode) of imaging. The B-mode is helpful to view the images in a two-dimensional plane, and the M-mode is useful to interpret ultrasound images along a single scan line. The M-mode is used to detect pneumothorax, pleural effusion and hydropneumothorax [7]. Recent studies have also shown that based on pleural and subpleural morphological features, M-mode ultrasonography is helpful to differentiate cardiac pulmonary edema from non-cardiogenic alveolar interstitial syndrome [8].

A high-frequency linear probe of 7.5-10 MHz can be used in thoracic imaging. In cases of obese patients with high adipose tissues, the tissue penetration can be limited with these high-frequency probes. Hence, in such patients, where the subcutaneous tissue is more than 5 to 8 cm, it is better to use a lower-frequency probe for better visualization [9]. The probe should be placed in the intercostal acoustic window in the longitudinal axis, parallel to the long axis of the patient. This allows clear visualization in the intercostal space. The probe should not be placed on the ribs as they will block the sound waves and create an acoustic shadow. LUS can be performed in the supine position, but lateral decubitus offers a better view of the dorsal regions of the lower lobes. However, it can be difficult to acquire a lateral decubitus position in critically ill patients. The lung is divided into six regions on the basis of anatomical landmarks (anterior and posterior axillary lines): the upper and lower parts of the anterior, lateral and posterior chest wall. On imaging, Merlin space is defined as the area located between the pleural lines and the shadow of the ribs and the bottom of the screen. Lung imaging is based on the relevant artifacts. All the lung signs arise at the level of pleura and can be static or dynamic. The first static artifact is the ‘A-line’. The A-line is a hyperechoic line generated parallel to the pleural line in the Merlin space. Another static artifact is the ‘B-line’. The B-line is a vertical artifact line that starts at the pleural line and spreads to the end of the screen. The B-line is also known as ‘Comet tail’. If we observe many B-lines in a single lung scan, then it is known as ‘Lung rockets’. Another form of vertical lines that simulate the B lines but arise from the subcutaneous tissues instead of the pleural lines is known as the ‘E-lines’ that are seen in the subcutaneous emphysema (Table 1). The pleural lines in the B-mode generate a ‘bat sign’. ‘Lung sliding’ is a dynamic sign that indicates the sliding of the visceral pleura over the parietal pleura. Lung sliding generates a ‘seashore sign’ in the M-mode. With the understanding of these lung signs in different modes, we can distinguish a normal lung from lung pathologies [5].

**Table 1:** The clinical description of different line patterns seen in lung ultrasonography

Line pattern	Clinical description
A line	A line is a static artifact generated parallel to the pleural line in the Merlin space. It is usually seen in normal lung ultrasonography.
B line	B line is generated as a vertical artifact, that begins at the pleural line and spreads to the edge of the screen. It is a pathologic sign seen in alveolar-interstitial syndrome.
E line	E lines are the vertical lines that arise from the subcutaneous tissue instead of the pleural lines and are seen in subcutaneous emphysema.
V line	In patients with pleural effusion or hemothorax, the V lines can be appreciated as the contour of vertebrae, when the probe is placed at the level of the diaphragm and aimed towards the spine in the supine position.

Bedside lung ultrasound in critically ill

Pneumothorax

On imaging in pneumothorax, the air between the parietal and visceral pleura reflects the ultrasound waves, thereby disrupting the B-line, but the A-lines remain intact. The bat sign is absent in the pneumothorax [10]. The presence of the A-line and the absence of lung sliding have a sensitivity and a negative predictive value of 100% and specificity of 96.5% in detecting the pneumothorax [11]. The ‘seashore sign’ seen in the M-mode is replaced by the ‘barcode sign’ or the ‘stratosphere sign’ in the pneumothorax. Another important sign ‘lung point’ is a dynamic sign. It is the point of contact between the lung and pleura in case of mild or moderate pneumothorax, where the reappearance of lung sliding and B-lines can be seen. The presence of a lung point is highly suggestive of pneumothorax. The lung point has a sensitivity of 66% and specificity of 100% in a prospective study with 44 out of 66 patients being diagnosed with pneumothorax [9]. LUS is also helpful to rule out pneumothorax after an invasive procedure or to detect the presence of pneumothorax after removing the chest tube [12-13].

Pleural Effusion/Hemothorax

Intrathoracic fluid in conditions like pleural effusion or hemothorax can be best visualized in the sitting position. However, in critically ill patients, it is difficult to prop them in a sitting position. Hence, these patients can be positioned in a heads up or a reverse trendelenburg position, in order to allow the fluid to settle at the lung base for better imaging. Simple effusions tend to settle at the posterior and inferior costophrenic angles. For better visualization of the effusion, a phased-array probe of a frequency of 3 to 5 MHz should be used. Intrathoracic fluid collections can be visualized as anechoic, echogenic or complex with internal septation. Transudate effusions observed in conditions like nephrotic syndrome, congestive heart failure and liver cirrhosis are always homogenous anechoic, whereas exudative fluid can be anechoic, echogenic or complex depending on the underlying pathology. On the other hand, blood is usually visualized as a heterogenous anechoic or hypoechoic collection [14]. There are three cardinal signs for the diagnosis of pleural effusion or hemothorax. In the B-mode, a small pocket of fluid between the lung and the chest wall is visualized as an anechoic quadrangle, with the base formed by the surface of the lung, the sides formed by the ribs and the top by the parietal pleura or the chest wall. The visualization of this quadrangle is known as the ‘quad sign’. In the M-mode, the ‘sinusoid sign’ is visualized. A bright sinusoid wave pattern can be seen, when the hyperechoic visceral pleura tries to approach the chest wall and recede during each respiratory cycle. Another sign recently appreciated in effusion or hemothorax is the 'vertebral line' (V-line). In the normal lung, when the probe is placed at the level of the diaphragm and aimed towards the spine, the posterior thoracic structures are not visualized because of the inflated lungs, but as we know fluid can act as an acoustic window, so when the probe is placed in the same position in patients with effusion or hemothorax, it is possible to visualize the contour of the vertebrae [15].

Lung Consolidation

Consolidations are appreciated as hyperechoic air bronchograms with a background of hypoechoic lung tissues [16]. The air bronchograms on LUS are specific for the diagnosis of consolidations and ventilator-associated pneumonia with a sensitivity of 100% and specificity of 60% [17]. The phased-array probes are preferable over the curvilinear probes for better visualization of the consolidation.

Interstitial Lung Disease

The deposition of collagen and fibrous tissue results in thickening of the interlobular septa in interstitial lung disease (ILD). These thickened septa cause the reflection of the ultrasound waves resulting in the visualization of diffuse B-lines or lung rockets. In early cases of ILD, the average distance between the B-lines of the thickened septa is roughly 3 mm and in advanced diseases, the distance is approximately 7 mm. LUS is helpful to detect the different stages of ILD [18].

Lung Abscess

LUS has diagnostic and therapeutic benefits in the detection and aspiration of the lung abscess. The lung abscess is visualized as a hypoechoic lesion with irregular margins seen as a hyperechoic ring [19]. 

Protocols for lung ultrasound in critically ill

The application of LUCI is explained in two protocols: the Bedside Lung Ultrasound in Emergency (BLUE) protocol for the diagnosis of acute respiratory failure and the Fluid Administration Limited by Lung Sonography (FALLS) protocol, for the management of acute circulatory failure.

The BLUE Protocol

The BLUE protocol was initiated for the early diagnosis of acute respiratory failure in critical care within a span of three minutes. This approach helps identify the diagnosis on the basis of pathognomic LUS signs for each disease. The following subcategory is included under this protocol:

A-profile is indicated by the presence of lung sliding with the presence of an A-line. It is observed in normal LUS. The A' profile is defined by the presence of A-lines without lung sliding. This profile is seen in pneumothorax. B profile includes lung sliding with numerous lung rockets, which indicates pulmonary edema. B' profile includes anterior lung rockets without lung sliding. This is usually seen in inflammatory interstitial syndrome (pneumonia). A/B profile includes unilateral lung rockets. C profile includes anterior lung consolidation indicating pneumonia. Postero-lateral alveolar pleural syndrome (PLAPS) includes posterior chest wall lung consolidations and pleural effusions. The A profile with no deep vein thrombosis (DVT) PLAPS (A-no-V-PLAPS) is specific for pneumonia. The BLUE protocol has an accuracy of 90.5 % to establish a diagnosis in acute respiratory failure [20].

**Figure 1:**
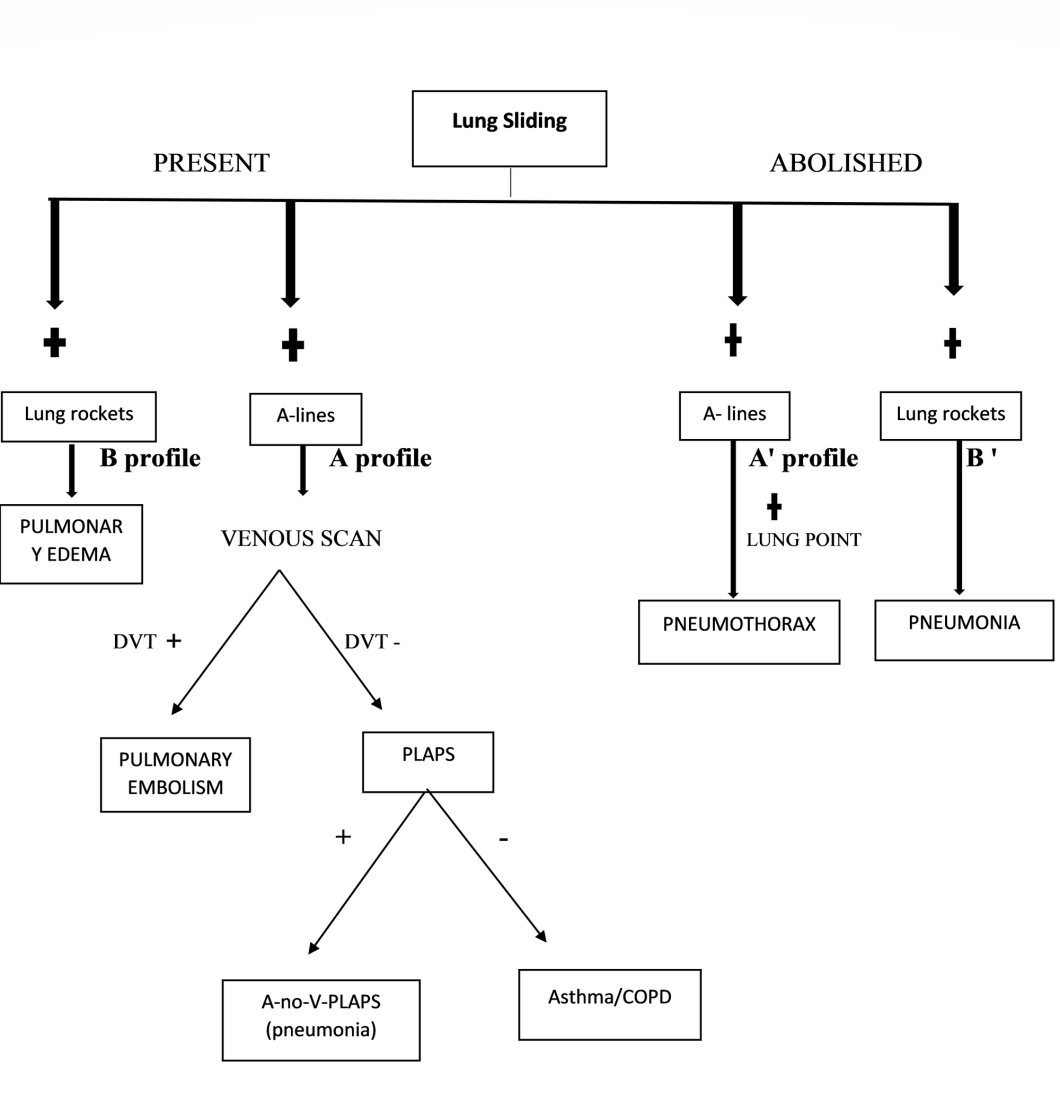
The protocol for Bedside Lung Ultrasonography in Emergency (Blue Protocol) DVT: deep vein thrombosis; PLAPS: posterolateral alveolar pleural syndrome; COPD: chronic obstructive pulmonary disease; A-no-V-PLAPS: A-profile with no DVT PLAPS

According to the BLUE protocol, the first thing to determine on lung imaging is the lung sliding. If the lung sliding is present, look for the A-lines. This defines the A-profile. The A-profile should be followed by a venous scan to determine DVT. If DVT is determined, then there are likely chances of having a pulmonary embolism. The specificity of DVT with A profile for pulmonary embolism is 99%. If the venous scan is negative, the presence of A-no-V-PLAPS is suggestive of pneumonia. If the venous scan and PLAPS points are both negative, then this nude profile suggests chronic obstructive pulmonary disease (COPD) or asthma. The presence of a B-profile is suggestive of pulmonary edema. The A' profile with the visualization of lung point is suggestive of pneumothorax. Lastly, the presence of the B' profile, A/B profile and the C-profile are highly suggestive of pneumonia (Figure 1) [21]. The accuracy of the BLUE protocol was further noted by Lichtenstein et al., wherein they determined the sensitivity and specificity of the B profile as 97% and 95%, respectively, the A profile with DVT as 81% and 99%, respectively and the A' profile with lung point as 88% and 100%, respectively [20].

The FALLS Protocol

The FALLS protocol aims to evaluate the acute circulatory shock in a critical care setting. The FALLS protocol follows the Weil classification of shock and helps rule out obstructive shock, cardiogenic shock, hypovolemic shock and septic shock in an emergency setting. Fluid overload can end in harmful consequences of pulmonary edema, which can be assessed on LUS. The initial step in this protocol is the evaluation of the pericardial effusion followed by the assessment of right ventricular volume and pneumothorax. This step rules out obstructive shock if no disorder is found on LUS. The next step is to determine the presence of B profile in order to rule out a cardiogenic shock. After this step, the fluid therapy is administered to determine hypovolemic shock. If the patient does not show improvement with the fluid therapy, fluids are discontinued and further analysis is resumed to determine the type of shock [19]. The major limitations of this protocol are that it does not address the complete hemodynamic assessment in critically ill patients. Further clinical trials are required to determine the accuracy and efficacy of this protocol.

## Conclusions

In recent years, there is a better understanding of the utility of LUS in critical care illness. This imaging modality is an essential tool for a diagnostic and therapeutic approach in emergency care and has the potentials to grow as the stethoscope of the 21st century. It is vital for hospitalists and intensivists to understand the role of LUS and unfold its utility in emergency and critical care settings.
